# Non-immune regulation of tertiary lymphoid structures in cancer

**DOI:** 10.3389/fimmu.2026.1947028

**Published:** 2026-09-18

**Authors:** Owen K. Challen, Evelyn Fitzsimons, Wyatt W. Anderson, Daniella Liao, Willie Wu, Vanessa G. P. Souza, Katya H. Bénard, Julia R. Naso, Spencer D. Martin, Wan L. Lam, Katey S. S. Enfield

**Affiliations:** 1 Basic and Translational Research, BC Cancer Research Institute, Vancouver, BC, Canada; 2 Biochemistry and Microbiology, University of Victoria, Victoria, BC, Canada; 3 Pathology and Laboratory Medicine, University of British Columbia, Vancouver, BC, Canada; 4 Interdisciplinary Oncology Program, University of British Columbia, Vancouver, BC, Canada; 5 Pathology and Laboratory Medicine, Vancouver General Hospital, Vancouver, BC, Canada; 6 Department of Pathology, Dalhousie University, Halifax, NS, Canada

**Keywords:** cancer metabolism, cancer-associated fibroblasts, high endothelial venules, immunotherapy response, spatial biology, tertiary lymphoid structures, tumor microenvironment

## Abstract

Tertiary lymphoid structures (TLS) are ectopic immune aggregates that form in response to chronic inflammation, and their presence across many solid tumors has been associated with improved survival and enhanced responses to immunotherapy. Although current models primarily characterize TLS through the lens of immune aggregation and organization, their formation also depends on reciprocal interactions with the non-immune tumor microenvironment. Fibroblasts, vasculature, extracellular matrix architecture, and tumor metabolism collectively influence TLS development, suggesting that these structures function as emergent tissue ecosystems rather than as immune aggregates alone. In this review, we summarize current evidence on how cancer-associated fibroblasts and the tumor vasculature regulate immune cell recruitment and organization through context-dependent expression of chemokines, adhesion molecules, structural barriers, and vascular niches, before considering how extracellular matrix remodeling and tumor metabolism further shape TLS neogenesis and maturation. Across these processes, non-immune components exhibit dual functions, promoting TLS formation in some contexts while reinforcing immune exclusion and antagonizing TLS in others. We further discuss current spatial technologies that enable in-depth quantification of TLS structure and maturity and clarify the contribution of non-immune components to TLS biology. Due to this increased appreciation of their biological relevance, we discuss the promise of non-immune components as rational targets for combination strategies to enhance TLS function and immunotherapy efficacy.

## 1 Introduction

Tertiary lymphoid structures (TLS) are organized ectopic immune aggregates resembling lymph nodes that form in response to chronic inflammatory conditions, including cancer (1). Across many solid tumors, the presence of TLS has been associated with improved survival and enhanced responses to immune checkpoint inhibitors (ICIs), demonstrating their prognostic and predictive relevance (2, 3). The current mechanistic model describes TLS as promoting anti-tumor immunity by creating niches for localized antigen presentation and lymphocyte maturation; however, these processes remain incompletely understood. TLS are defined across a spectrum of maturation. While definitions of maturity vary across studies, descriptions generally range from relatively poorly organized lymphoid aggregates or immature TLS, to increasingly organized structures with defined T- and B-cell zones, follicular dendritic cell (DC) networks, and specialized vasculature indicative of germinal centers (3–5). Although TLS are often viewed through an immunological lens, their development also relies on reciprocal interactions between immune and non-immune components of the tumor microenvironment (TME). Beyond leukocyte recruitment and activation, TLS formation requires structural support, chemokine gradients, vascular remodeling, and extracellular matrix (ECM) organization, processes that depend on sustained coordination within the TME (**Figure 1**). In this review, we highlight how non-immune components of the TME can both promote and constrain TLS development, discuss the spatial technologies enabling their study, and consider how these processes might be therapeutically exploited to enhance anti-tumor immunity.

**Figure 1:**
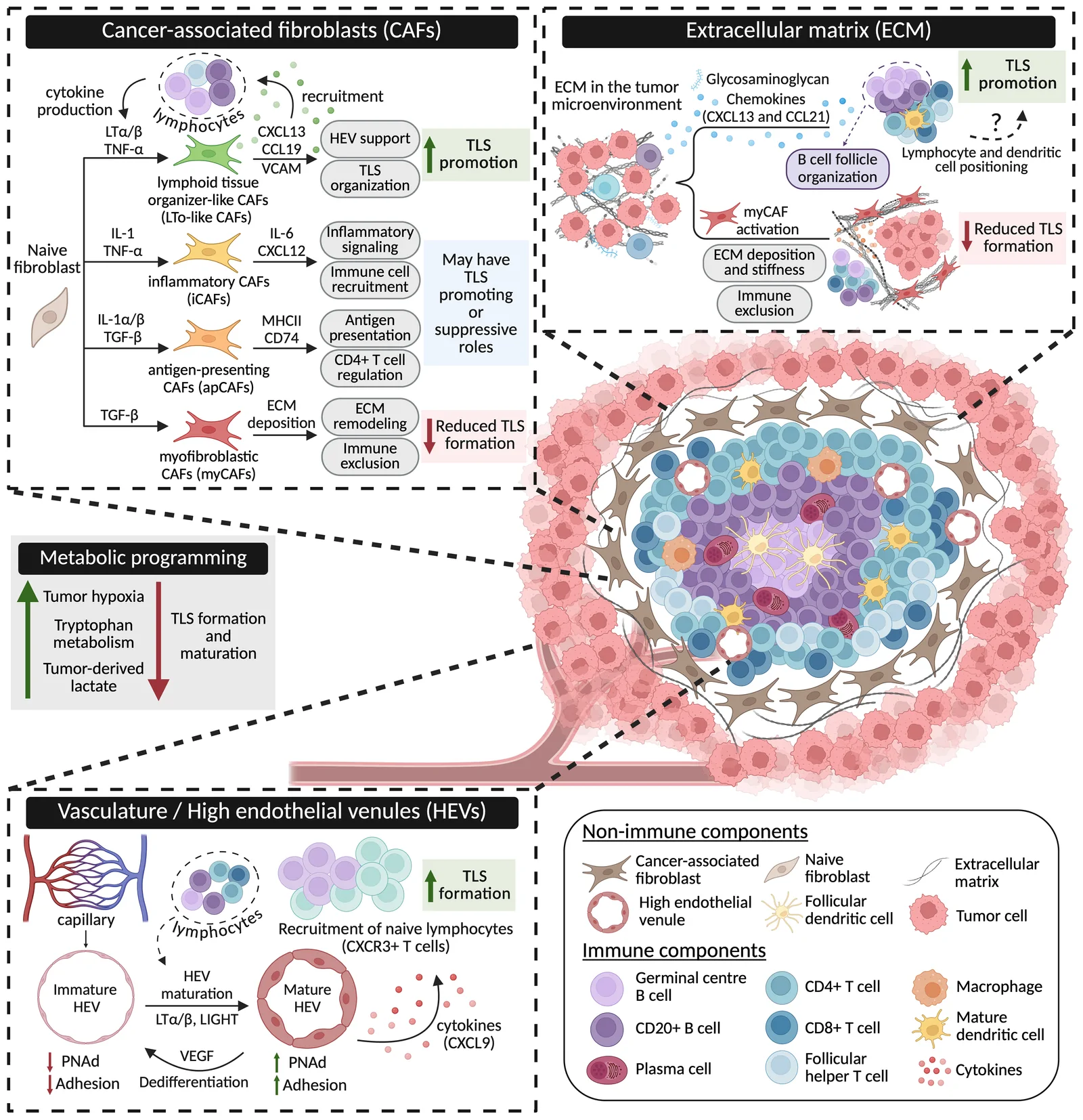
Non-immune regulation of tertiary lymphoid structure (TLS) formation and maturation within the tumor microenvironment (TME). Schematic of the TME showing how cancer-associated fibroblasts, extracellular matrix, high endothelial venules, and metabolic programming promote or impair TLS formation, with linked panels connecting each stromal and vascular process to TLS development and maturation. This figure was created in BioRender (https://BioRender.com/bkp495o) and is licensed under CC BY 4.0.

## 2 Cancer-associated fibroblasts as dual regulators of TLS formation

Cancer-associated fibroblasts (CAFs) are key regulators of the TME, shaping tumor architecture through ECM remodeling and reciprocal interactions with immune cells (6, 7). Their functions are highly heterogeneous and context-dependent, varying with tissue of origin, tumor type, and local signaling cues, resulting in dynamic cell states that can either promote or suppress TLS development and maturation (6, 8, 9) (**Figure 1**). Importantly, these CAF states should not be viewed as mutually exclusive or stable cell populations, but rather as dynamic states that can be shaped by tissue context and local signaling cues. Recent spatial and single-cell profiling studies have identified multiple CAF states, some of which appear to play opposing roles in TLS formation (**Supplementary Table 1**). While some consensus exists on broad defining features, studies vary in the technologies and phenotypic markers used to study these CAF states, reflecting a rapidly evolving field of study.

Our understanding of TLS-associated stromal populations is emerging, in part, from established knowledge of stromal biology in secondary lymphoid organs (SLOs). Lymphoid tissue organizer (LTo) cells and fibroblastic reticular cells (FRCs) are stromal fibroblast populations that are involved in the development and maintenance of SLOs (10, 11). LTo cells contribute to lymphocyte recruitment during SLO development, while mature FRCs, including T-cell zone reticular cells, maintain the stromal networks that support lymphocyte organization in mature SLOs through chemokines such as CCL19, CCL21 and CXCL13 (10–12). A study using cell fate mapping in lung cancer identified tumor-associated FRCs that organize T-cell niches and support lymphoid aggregation (13). CAFs with LTo-like features acquire characteristics and stromal programs similar to those of the fibroblast populations involved in SLO regulation, suggesting a potential mechanistic framework for TLS formation (9, 10).

Fibroblasts with LTo-like characteristics have been identified within multiple solid tumor types, with studies in melanoma and pancreatic cancer suggesting that inflammatory cytokines (LTα1β2, TNF-α) promote this lymphoid-organizing phenotype (9, 14). In murine melanoma models, intraperitoneal TMEs induced fibroblasts expressing LTβR, TNFR1, ICAM-1, and VCAM-1 that were associated with TLS formation, whereas subcutaneous TMEs generated FAP^+^ pro-tumor CAFs lacking lymphoid-organizing traits (9). In murine pancreatic cancer models, TLS-rich regions were found to contain CAF networks expressing VCAM-1 and secreting CXCL13 and CCL19 (14). Similar CXCL13^+^ fibroblasts have been identified in nasopharyngeal carcinoma, where they localized in TLS-associated regions and were associated with B-cell recruitment and germinal center formation (15). In penile squamous cell carcinoma, CAFs expressing CCL21, key for lymphocyte recruitment and SLO formation, were spatially enriched within mature TLS and adjacent to B-cell rich zones (10, 11, 16). Together, these findings support a model in which LTo-like fibroblasts recruit lymphocytes, while lymphocyte-derived LTα1β2 and TNF-α signaling reinforces the LTo-like phenotype, potentially creating a positive feedback loop promoting TLS formation and maturity.

In contrast to LTo-like fibroblasts, inflammatory CAFs (iCAFs) represent an inflammation-driven state whose role in TLS formation appears less direct. Induced by cytokines such as IL-1 and TNF-α, iCAFs secrete inflammatory mediators including IL-6 (17). Although previously described as residing distal to the tumor nest, an emerging spatial transcriptomics study of intrahepatic cholangiocarcinoma identified iCAFs adjacent to aggregated intratumoral TLS, where they co-localized with CXCL12^+^ macrophages (8, 18). CAF composition also shifted during TLS development, with iCAF signaling enriched in early immature TLS and IFN-producing CAFs becoming more prominent during maturation (18). These findings suggest that iCAFs contribute to early TLS-associated stromal signaling but are unlikely to serve as the principal structural organizers.

Antigen presentation represents a less understood mechanism through which CAFs may shape adaptive immunity within TLS. Antigen-presenting CAFs (apCAFs) are defined by MHC-II expression and can be induced by signals including IL-1α/β and TGF-β (19). A fibrocyte-like apCAF population was recently identified as enriched within TLS across multiple tumor types; however, this spatial association does not establish a role for apCAFs in TLS formation or maturation and may instead reflect the inflammatory environment associated with established TLS, including signals derived from immune cells such as macrophages (20). Consistent with a potential role in local T-cell activation, fibroblast-specific ablation of MHC-II induces a hypometabolic state in CD4^+^ T-cells and accelerates tumor growth (21). These findings suggest that apCAFs can influence local CD4^+^ T-cell responses, although the timing of their induction and whether they directly promote TLS maturation or T-cell dysfunction remains unresolved. Importantly, the relationship between apCAFs and TLS may be reciprocal, whereby inflammatory and immune-cell-derived signals within developing or established TLS induce an antigen-presenting phenotype in local fibroblasts, while apCAFs may in turn modulate local T-cell responses. Thus, the enrichment of apCAFs around TLS should not be interpreted as evidence of a causal role in TLS development, and further studies are required to distinguish whether apCAF accumulation is a driver, consequence, or component of the inflammatory processes associated with TLS formation.

Myofibroblastic CAFs (myCAFs) are a conserved fibroblast phenotype, characterized by high ɑSMA expression and a contractile, TGF-β-driven state (7, 22). Compared with other CAF subsets, myCAFs express fewer immune-recruiting cytokines and instead deposit dense ECM, contributing to immune exclusion (23, 24). Accordingly, they are associated with TGF-β-rich immunosuppressive TMEs that typically lack TLS (14, 25). In pancreatic ductal adenocarcinoma, TGF-β suppresses LTβR/TNFR1 signaling, diverting fibroblasts from a TLS-supportive LTo-like phenotype towards myCAFs (14). Collectively, these findings support a role for CAF heterogeneity in shaping TLS-permissive versus TLS-restrictive stromal niches, though the causal contributions of individual CAF states remain incompletely defined.

## 3 High endothelial venules as gatekeepers of lymphocyte recruitment

Although CAFs provide the stromal framework for immune organization, TLS formation also requires recruitment of lymphocytes from circulation. High endothelial venules (HEVs) are specialized endothelial structures, typically characterized by peripheral node addressins (PNAd) expression (26). While normally found in lymph nodes, similar PNAd vascular structures occur in tumors, where they are considered markers of mature TLS and directly bind to CD62L found on naive and memory lymphocytes (4, 27, 28). Early lymphoid aggregates can form before the acquisition of a mature HEV phenotype; however, as TLS develop, HEVs emerge and facilitate continued lymphocyte recruitment, thereby supporting further TLS maturation. Consistent with this stage-dependent role, murine intravital microscopy showed that tumor-associated HEVs (TA-HEVs) actively mediate lymphocyte entry into tumors (28).

In sarcoma and colorectal carcinoma models, TA-HEVs were the primary sites of lymphocyte arrest and extravasation within the TME, supporting recruitment both at baseline and following ICI (28). Beyond PNAd, this process is mediated by adhesion molecules including ICAM-1 and VCAM-1, which promote lymphocyte extravasation (29, 30). In human gastric cancer, HEVs expressing these molecules recruited and activated CXCL13-producing T-cells through the CXCL13/ACKR1 pathway, potentially driving TLS formation through subsequent B-cell interactions (31). Consistent with this, increased HEV density in human cancers correlates with enhanced T- and B-cell infiltration and improved ICI response (28, 32). Together, these observations highlight HEVs as the highways sustaining lymphocytic influx central to TLS establishment and development.

HEVs must also differentiate and be maintained within the tumor, a process dependent on LTβR signaling, determined through experiments using a decoy receptor to LTβR (28, 33). LTβR ligands, including LTα1β2 and LIGHT, are primarily expressed by innate lymphoid cells and lymphocytes, suggesting these cells contribute to HEV differentiation in situ (34). Fate-mapping studies in murine tumor models treated with immunomodulatory antiangiogenic therapy show that HEVs arise from pre-existing post-capillary venules, with transdifferentiation requiring continuous local LTβR signaling for induction and maintenance (30, 35). Mature PNAd-rich HEVs rapidly dedifferentiate following LTβR inhibition or depletion of DCs (33, 35, 36). In contrast, VEGF signaling through the VEGF-D/VEGFR2 axis promotes HEV dedifferentiation and impairs lymphocyte recruitment in tumor-draining lymph nodes (37). Whether a similar mechanism occurs directly in tumors remains to be determined. Together, these findings suggest a reciprocal process in which HEVs recruit lymphocytes, while recruited lymphocytes and DCs reinforce the HEV phenotype.

Specialized HEV subsets with distinct roles in TLS organization have also been identified. In nasopharyngeal carcinoma, an interferon-responsive CXCL9-secreting HEV subset recruited CXCR3^+^CD4^+^ T-cells into TLS, while spatial analysis in non-small cell lung cancer (NSCLC) identified ID1-high HEVs associated with TLS maturation and improved ICI response (38, 39). Collectively, these findings establish HEVs as dynamic, signal-dependent gatekeepers of lymphocyte entry into the TME that emerge during TLS development and reinforce maturation through further lymphocyte recruitment.

## 4 The extracellular matrix as an instructive TLS niche

Fibroblast identity and HEV differentiation are shaped by the surrounding physical and biochemical environment, extending regulation of TLS formation to the ECM itself. Beyond serving as a scaffold, the ECM influences immune cell migration, stromal cell behavior, and chemokine localization required for TLS formation. In SLOs, glycosaminoglycans within the ECM bind chemokines including CXCL13 and CCL21, generating immobilized gradients that direct lymphocyte and DC positioning and contribute to B-cell follicle organization, highlighting how matrix architecture can regulate immune cell compartmentalization (40–42). Although these mechanisms provide a plausible framework for how matrix organization could influence TLS development, their direct contribution to TLS formation in tumors remains incompletely established. Thus, ECM remodeling may influence TLS indirectly by altering immune cell access and the spatial distribution of chemokine cues required for lymphoid organization.

The link between ECM-associated cellular regulation programs and tumor TLS is more directly exemplified in NSCLC. In this setting, the actin regulatory protein hMENA undergoes tissue-specific splicing in response to cues including TGF-β, generating two functionally opposing isoforms (43). In CAFs, the hMENAΔv6 isoform promotes fibronectin deposition while suppressing LTβR expression, LTβR-associated signaling and CXCL13 production. In contrast, the epithelial isoform hMENA11a, expressed in tumor cells, reduces fibronectin deposition while promoting LTβR expression and CXCL13 production. Importantly, these molecular patterns were associated with TLS localization in NSCLC: tumors with high hMENA11a, low CAF hMENAΔv6, and low stromal fibronectin were more frequently characterized by intratumoral TLS, whereas higher stromal fibronectin was associated with peritumoral TLS (43). These findings provide evidence linking cellular regulation of the ECM to the spatial localization of tumor-associated TLS, although they do not establish that fibronectin remodeling alone is sufficient to initiate TLS formation.

Beyond ECM composition, the physical properties of the matrix also regulate stromal phenotypes through mechanotransduction pathways. Matrix stiffness is sensed via integrins and focal adhesions, activating signals that influence fibroblast differentiation, inflammatory gene expression, and further ECM remodeling (44). This reinforces myCAF activation, creating a feed-forward loop that promotes tissue stiffening and immune exclusion (45, 46). In this context, increased matrix stiffness may restrict immune cell access to tumor parenchyma and thereby influence where TLS can form or mature, but whether matrix stiffness directly regulates TLS initiation or maturation remains uncertain. Together, these findings support a model in which ECM remodeling can influence TLS organization primarily through its effects on chemokine distribution, stromal phenotype, and immune cell access, while the direct causal contribution of matrix composition and stiffness to TLS initiation and maturation remains to be established.

## 5 Metabolic reprogramming of the tumor microenvironment shapes TLS development

In addition to stromal and vascular signals, the metabolic landscape of the TME influences TLS development by shaping immune cell survival and activation. In NSCLC, tumor hypoxia has been associated with reduced TLS activation, with hypoxia scores in TLS correlating positively with Treg proportions and negatively with effector CD4^+^ populations (47). Similarly, hypoxic germinal centers in tumor-draining lymph nodes were found to negatively regulate humoral immune responses in preclinical models (48). This contrasts with healthy lymph nodes, where hypoxia promotes germinal center functions including class-switching and plasma cell differentiation, while reversal of hypoxia reduces germinal center immune cell populations (49). These findings suggest that hypoxia regulates TLS development in a context-dependent manner, likely through interactions with the broader metabolic and immunosuppressive landscape of the TME rather than oxygen availability alone.

Metabolic byproducts may also influence TLS development. In triple-negative breast cancer models, tumor-derived lactate impaired the chemotaxis of B-cells from cancer-associated lymph nodes into tumors, preventing TLS formation despite strong CXCL13 and CCL21 signaling. Inhibiting lactate production alongside chemokine delivery restored B-cell infiltration, while combining this with a CD40 agonist increased TLS number, size, and maturity (50).

Altered amino acid metabolism may further reinforce immunosuppressive conditions that limit TLS formation. In hepatocellular carcinoma, increased TDO2-mediated tryptophan metabolism elevated local kynurenine concentrations within the TME, generating an immunosuppressive niche that restricted TLS maturation (51). Pharmacological inhibition of this pathway restored intratumoral TLS maturation in preclinical models and enhanced responses to ICI (51). Competition for arginine and glutamine similarly constrains T-cell survival and effector function within the TME, although whether this directly affects TLS formation and maturation, rather than T-cell function more broadly, remains unclear (52, 53). Together, these findings indicate that hypoxia, amino acid availability, and metabolic byproducts shape TLS formation and support immunosuppression in the TME.

## 6 Spatial technologies for characterizing non-immune regulation of TLS biology

Advances in spatial technologies and computational pathology have transformed our ability to interrogate the TME (**Supplementary Table 2**). We can now quantitatively characterize the composition, structure, and localization of TLS, enabling detailed investigation into how their maturity and positioning within the tumor architecture shapes disease progression and ICI response.

TLS have long been identified and studied using conventional staining methods such as hematoxylin and eosin (H&E), May-Grünwald-Giemsa, and colloidal gold. These methods have contributed to grading of TLS along a spectrum of maturity, from lymphoid aggregates that may represent early immature TLS, to fully mature TLS identified by the presence of a defined germinal center and based on the topographical organization of lymphocytes and macrophages, degree of angiogenesis, and presence of plasma cells (54–58). Emerging machine learning models have begun to automate this process, enabling high-throughput detection and grading of TLS from H&E images (59). Signatures associated with immune infiltration of the TME and components of TLS have also been derived from bulk RNA-sequencing and flow cytometry in multiple cancers (60–65). These studies have provided insights into TME cell composition and defined protein- and RNA-based signatures associated with patient treatment outcomes; however, they fail to capture the spatial information and phenotypic detail needed for in-depth characterization of TLS. Multiplexed antibody-based methods, including multiplex immunohistochemistry and immunofluorescence, have begun to address this limitation through simultaneous detection of an increasing number of cell phenotypes (57, 58, 66–68). These technologies have facilitated evolving definitions of TLS maturity, with features such as B- and T-cell compartmentalization, follicular DCs, CD21 and CD23 staining, cytokine expression, and proliferative marker Ki67, as well as HEVs, described as indicators of mature TLS (1, 4, 9, 15, 16, 18, 31, 38, 39). In addition to maturity, TLS localization within the TME has been associated with patient outcome and treatment response (1). TLS developing in intratumoral, peritumoral, or marginal sites may hold distinct biological significance and warrant annotation in future studies. Overall, developments in highly multiplexed immunofluorescence technologies have refined our understanding of TLS biology and begun to connect maturation state and localization to treatment outcomes across multiple cancers (4, 54, 56, 68–72).

Despite these advances, several questions central to TLS biology remain unresolved by current multiplexed imaging approaches. The proteomic depth achievable by antibody panels is inherently limited, constraining unbiased discovery of novel markers and functional states. Curated antibody panels cannot capture the broader transcriptional and proteomic programs that remain relatively uncharacterized across tumor, immune, and stromal populations within and surrounding TLS. These panels are similarly limited in their ability to localize secreted or rapidly degraded molecules, such as cytokines and chemokines, that orchestrate lymphocyte recruitment and organization. To further our understanding of the biological differences in TLS based on their localization, as well as how their surrounding environment regulates TLS formation and maturity, deeper characterization is required.

The next generation of spatial technologies, spatial transcriptomics, and deep visual proteomics (DVP), are primed to support discovery in these areas. Spatial transcriptomics measures expression of hundreds to thousands of genes within intact tissue, enabling deep phenotyping of TLS and the associated TME (73, 74). This allows for the identification of transcriptional programs associated with TLS-restrictive or permissive environments, deeper phenotyping of TLS maturity and functional states, and ultimately, features associated with patient outcome including treatment response. Because morphologically irregular cells, such as CAFs, are difficult to segment from transcriptomic data alone, spatial transcriptomics may be paired with multiplexed immunofluorescence to help resolve cell boundaries and improve segmentation accuracy (31). This approach further enables the identification of cell types of origin for transcripts encoding secreted or rapidly degraded molecules. DVP takes a complementary approach, also beginning with multiplexed immunostaining, to identify and phenotype cells for targeted laser microdissection (**Figure 2**). DVP combines artificial intelligence-driven image analysis and ultra-high-sensitivity mass spectrometry to generate detailed untargeted proteomic profiles of spatially defined cell populations, addressing proteomic depth limitations of multiplexed imaging technologies (75). DVP is also well suited to identifying receptor-ligand pairs relevant to TLS regulation, including the discovery of novel interactions among CAF, endothelial, and immune populations. This includes PD-1:PD-L1 interactions, central to regulating B-cell maturation in germinal centers, allowing direct assessment of how ICIs modulate these processes within TLS and their surrounding niche (76). Together, spatial transcriptomics and DVP provide an unprecedented view of cellular heterogeneity and functional states, offering the resolution required to understand how TLS are initiated and sustained as well as their phenotypic diversity (77). By moving beyond descriptive characterization, these technologies will allow mechanistic investigation of how stromal, vascular, and ECM components orchestrate TLS maturation within the TME, ultimately providing new insights into tumor immunology and patient responses to ICIs.

**Figure 2:**
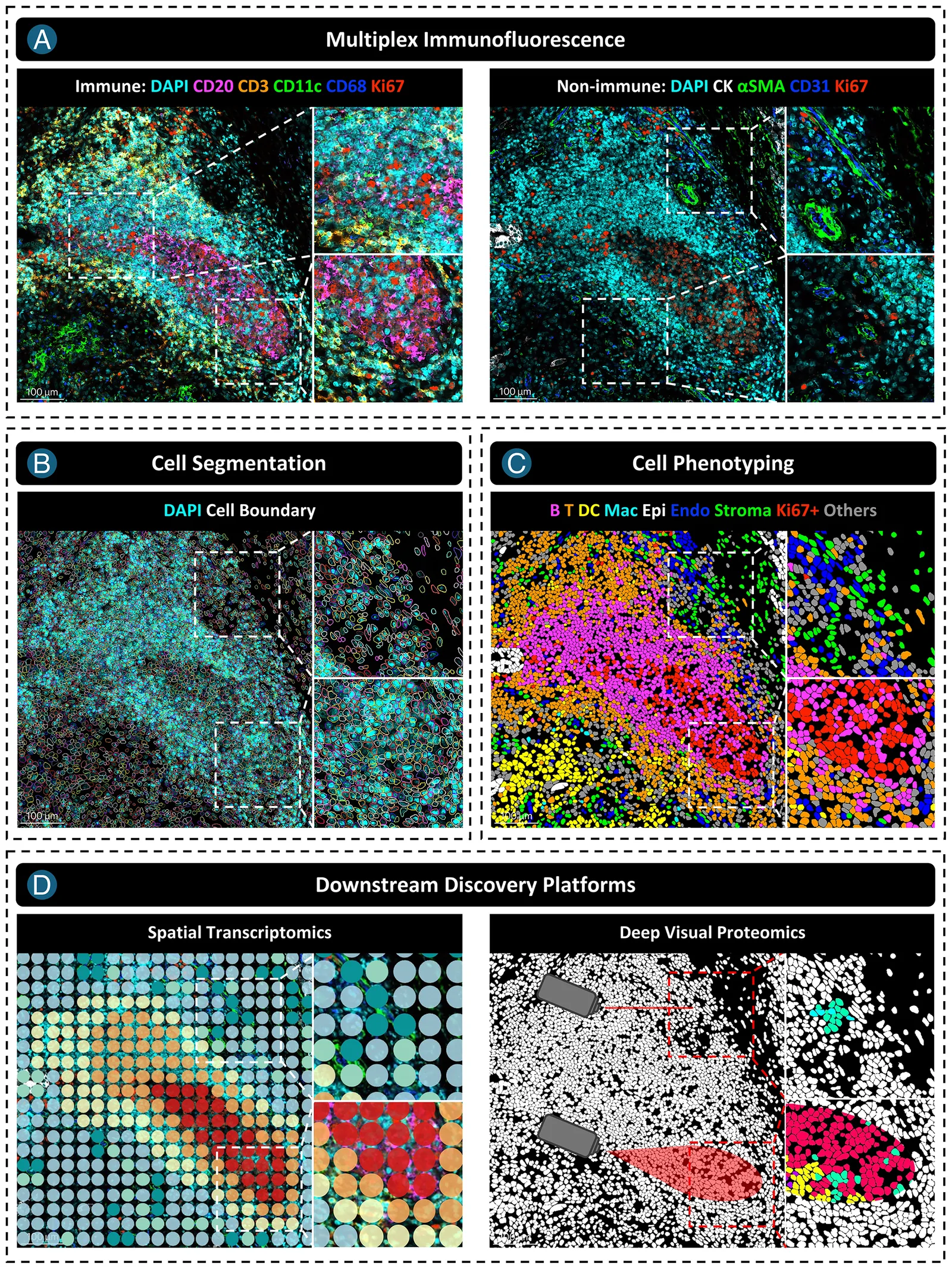
Advanced spatial technologies enable comprehensive characterization of tertiary lymphoid structures (TLS) and their surrounding environment at single-cell resolution. **(A)** Illustrative example of a mature TLS in a solid tumor with both immune (left) and non-immune cell markers (right) captured in a multiplex immunofluorescence experiment. **(B)** Single-cell segmentation delineates individual cellular boundaries. **(C)** Cells are classified based on multiplex marker expression profiles into B cells (B), T cells (T), dendritic cells (DC), macrophages (Mac), epithelial cells (Epi), endothelial cells (Endo), stromal cells (Stroma), proliferating Ki67^+^ B cells, and other cell populations. Multiplexed immunofluorescence data can be generated on its own or in combination with **(D)** downstream discovery platforms to aid assignment of transcripts to cells using spatial transcriptomics or to select single-cell phenotypes or structures of interest with Deep Visual Proteomics. Scale bar = 100 µm.

## 7 Translational opportunities for targeting the non-immune TME to promote TLS function

Although TLS have emerged as promising predictive biomarkers of response to ICIs, their translational significance may extend beyond biomarker applications. As critical regulators of TLS formation and maturation, CAFs, vascular networks, and ECM architecture represent attractive therapeutic targets, offering a route to promote TLS function and improve ICI responses.

Among current therapeutic strategies, vascular normalization provides one of the clearest examples of how targeting non-immune TME components may influence TLS biology. Tumor-associated blood vessels are structurally and functionally abnormal, restricting lymphocyte trafficking, promoting hypoxia, and limiting productive immune niches. Inhibition of VEGF signaling restores vessel structure and function, improving perfusion and immune cell access to tumors (78, 79). Dual inhibition of VEGF-A and angiopoietin-2 similarly enhances T-cell infiltration and is associated with increased HEV formation (80), while combining anti-angiogenic therapy with ICIs increases HEV density and local immune activation, supporting the concept that vascular remodeling can facilitate the establishment of organized intratumoral immune niches (81). Clinically, anti-VEGF-ICI combinations have demonstrated improved outcomes in several malignancies in which ICIs are already established treatment options, including renal cell carcinoma, hepatocellular carcinoma, and NSCLC (82–84). These benefits are generally attributed to improved immune infiltration following vascular normalization, but the accompanying induction of HEVs and potential promotion of TLS may be an underappreciated contributor to therapeutic efficacy. This warrants longitudinal assessment of TLS presence, localization, and maturity in patients receiving vascular-targeted therapies.

Targeting CAFs presents a more complex challenge, since the same heterogeneity that shapes TLS formation also complicates therapeutic manipulation. Early approaches sought to broadly deplete stromal fibroblasts; however, because certain subsets such as LTo-like CAFs actively support TLS formation, indiscriminate depletion risks disrupting anti-tumor immunity (85, 86). Current approaches therefore favor selective inhibition of CAF-derived immunosuppressive signals, such as TGF-β and CXCL12, which contribute to immune exclusion and impaired anti-tumor immunity. A fibroblast-associated TGF-β signature correlated with CD8^+^ T-cell exclusion and resistance to PD-L1 blockade in metastatic urothelial cancer, with combined TGF-β/PD-L1 inhibition restoring T-cell infiltration in preclinical models (87). Similarly, inhibition of CAF-derived CXCL12 synergized with PD-L1 blockade in pancreatic cancer by overcoming stromal immune exclusion (88), while distinct myCAF subsets suppressed CD8^+^ T-cell infiltration and conferred resistance to ICI in breast cancer (89). Current ICIs used in the clinic have been shown to reprogram CAFs. For example, anti-PD-1 treatment in breast cancer was associated with molecular reprogramming of CAFs, reducing ECM- and TGF-β-associated signaling while increasing immune-supportive signaling (90). Whether this reprogramming reflects a shift toward TLS-supportive CAF states, and whether it directly enhances TLS formation or maturation, remains an open question.

The multiple roles of the ECM in chemokine retention, cellular migration, mechanotransduction, and stromal cell differentiation have prompted interest in matrix-targeted therapies as adjuncts to ICIs. Inhibition of LOX-mediated collagen cross-linking reduces matrix stiffness, focal adhesion formation and tumor progression, while targeting focal adhesion kinase, a key mediator of integrin signaling, decreases stromal fibrosis, enhances CD8^+^ T-cell infiltration and sensitizes pancreatic tumors to PD-1 blockade (91, 92). Enzymatic depletion of hyaluronan similarly remodels the tumor stroma and may improve immune cell access by reducing physical barriers within the TME (93). However, whether matrix-normalizing strategies directly promote TLS initiation or maturation remains largely unexplored. Importantly, although these approaches can enhance immune cell infiltration and remodel the TME, their ability to directly induce or promote the maturation of functionally competent TLS remains unproven, as TLS-related endpoints have not been routinely assessed in these studies.

These translational strategies share a common limitation: the presence of immune infiltrates or TLS alone does not invariably predict responsiveness to ICIs (94, 95). This variability likely reflects the marked structural and functional heterogeneity of TLS, as well as the influence of the surrounding TME. Dysfunctional stromal programs, such as fibrotic ECM, aberrant vasculature, or immunosuppressive CAF populations may limit the functional maturation of TLS, such that their presence does not necessarily translate into effective anti-tumor immunity. In addition, tumor-intrinsic and microenvironmental immunosuppressive pathways can undermine anti-tumor immunity despite the presence of TLS, highlighting that the functional maturity and stromal context of TLS may be as important as their presence (96). Therapeutic manipulation of CAFs, vasculature, and ECM may therefore not only induce TLS but also promote functionally competent immune niches capable of enhancing responses to ICIs and other immunotherapies, including cancer vaccines and adoptive cell therapies.

## 8 Conclusions and future directions

Non-immune stromal, vascular, and matrix components play coordinated and necessary roles in TLS formation. CAFs establish stromal networks and chemokine gradients that organize immune cells, either supporting or suppressing TLS formation. HEVs regulate lymphocyte access through specialized adhesion molecules, while the ECM provides the biochemical and biomechanical framework that supports TLS structure and function. Indeed, successful TLS formation depends on coordinated interactions between immune and non-immune components of the TME. Evolving spatial technologies will help to define the mechanisms through which the TME regulates TLS formation, characterize the trajectory of TLS maturation, and clarify the functional importance of TLS localization within the tumor. These advances may lead to the discovery of novel targets to improve ICI efficacy and cancer patient outcomes.
